# A new species of *Myiocephalus* Marshall (Hymenoptera, Braconidae, Euphorinae) from China

**DOI:** 10.3897/zookeys.933.49607

**Published:** 2020-05-18

**Authors:** Jun Li, Cornelis van Achterberg, Min-Lin Zheng, Jia-Hua Chen

**Affiliations:** 1 Beneficial Insects Institute, Fujian Agriculture & Forestry University, Fuzhou, Fujian 350002, China Fujian Agriculture & Forestry University Fuzhou China; 2 State Key Laboratory of Rice Biology, Ministry of Agriculture Key Lab of Agricultural Biology of Crop Pathogens and Insects, and Institute of Insect Sciences, Zhejiang University, Hangzhou 310058, China Zhejiang University Hangzhou China

**Keywords:** identification, key, *
Myiocephalus
*, taxonomy

## Abstract

A new species of the genus *Myiocephalus* Marshall, 1898, *M. cracentis* Li, **sp. nov.** from the Palaearctic (China, Ningxia, Hubei), is described and illustrated. A key to known species of *Myiocephalus* is provided. *Myiocephalus boops* (Wesmael, 1835), is a new record for Jilin province (NE China).

## Introduction

Euphorinae (Hymenoptera, Braconidae) is a large subfamily of endoparasitoid wasps with more than 1,270 described species worldwide (Yu et al. 2016). Their morphology varies greatly and this is quite unusual for a single subfamily. This highly polymorphic subfamily is characterized by only one derived character state: the postero-distally open first subdiscal cell of the fore wing because fore wing CU1b is absent (Shaw 1985; Tobias 1986). The other general morphological character of Euphorinae is the often, but not always, bent fore wing vein SR1+3-SR, and a more or less specialized scape, eyes, clypeus, mandible, fore leg, first metasomal tergite, and ovipositor. Stigenberg et al. (2015) divided this subfamily into 14 tribes and 52 genera. Chen and van Achterberg (2019) recognized two additional tribes, Eadyini and Proclithrophorini, of which the latter had been included in the Townesilitini by Stigenberg et al. (2015) on the basis of their concatenated molecular data (18S, 28S, CAD, and COI). However, the morphology of Proclithrophorini conflict this synonymy and its position remains unresolved. *Myiocephalus* is the only genus of the MyiocephaliniChen and van Achterberg (2019), which is associated with ant nests of the genus *Formica* but has never been reared (Donisthorpe 1927).

The genus *Myiocephalus* Mashall (recognized as *Loxocephalus* Forster) was first placed in its own tribe as Loxocephalini by Shaw (1985). *Myiocephalus* is the sister group of the lineage comprising *Comsmophorus* + Syntretini supported by morphological characters: mesonotum shiny and notauli absent; scutellar furrow without cross-carinae; and dorsope absent (Shaw 1985). Stigenberg et al. (2015) supported the Syntretini as the sister tribe to the Myiocephalini on the basis of their concatenated molecular data (18S, 28S, CAD, and COI) and unique morphological characters (the bulging eyes and smooth mesosoma). There are only two other known tribes of euphorine wasps (Syntretini and Neoneurini) attacking Hymenoptera except Myiocephalini. Myiocephalini is more closely related to Syntretini, based on both morphological and molecular evidences.

The genus *Myiocephalus* Marshall is, although they are rare in collections, one of the most distinctive euphorine genera with its strongly transverse (and females anteriorly more or less concave) head, and elongate first metasomal tergite with very large laterope and compressed metasoma. Four species of the genus *Myiocephalus* are currently known: *M. boops* (Wesmael, 1835); *M. niger* Fischer, 1957; *M. laticeps* (Provancher, 1886); and *M. zwakhalsi* van Achterberg, 2019 (Tan et al. 2019).

The first author examined the collections applying the key of Tan et al. (2019) and discovered a new species than it was confirmed by second author and is described below.

## Materials and methods

Studied material was selected from the entomological collections of the Beneficial Insects Institute, China (BIIC). The specimens were collected using a sweep net. All specimens studied are deposited in BIIC.

Specimens were examined using a Zeiss Stemi 2000 stereomicroscope. The photographs were taken with a computer-connected Leica DFC450 digital camera mounted on a Leica M205C stereo microscope. All images were further processed using minor adjustment in Adobe Photoshop CC, such as image cropping and rotation, adjustment of contrast and brightness levels, color saturation, and background enhancement.

The terminology used for measurements and descriptions of morphological characters follows van Achterberg (1988, 1993). The veins of the fore wing are illustrated on Figure 20.

## Taxonomic accounts

### Myiocephalus

Taxon classificationAnimaliaHymenopteraBraconidae

Marshall, 1898

65F9521B-239A-518B-9344-83A045FA0BA3

Loxocephalus Foerster, 1863: 252. Type species (by monotypy): Loxocephalus longipes Foerster, 1863 [= Myiocephalus boops (Wesmael, 1835)]. Unavailable name.
Myiocephalus Marshall [in André], 1898: 218; Chen and van Achterberg 1997: 74; Belokobylskij 2000: 372. Type species (by monotypy): Microctonusboops Wesmael, 1835.
Spilomma Morley, 1909: 211. Type species (by monotypy): Spilomma falconivibrans Morley, 1909 [= Myiocephalus boops (Wesmael, 1835)]. Synonymized with Myiocephalus Marshall by Muesebeck (1936).

#### Diagnosis.

Laterope large, deep and submedially situated in slender first tergite; head in dorsal view strongly transverse and usually slightly concave anteriorly; eyes enlarged and protruding; clypeus rather narrow; scapus slightly or not enlarged and subequal to or shorter than third antennal segment; maxillary palpi with five segments, labial palpi with three segments; vein 1-SR+M of fore wing absent; vein 1-R1 longer than pterostigma; vein M+CU1 largely unsclerotized; middle and hind legs elongated; metasoma of ♀ strongly compressed with fifth sternite of ♀ finger-like protruding posteriorly; hypopygium of ♀ with long setae apically or hypopygium medially membranous. (Tan et al. 2019).

#### Distribution.

Nearctic, Palaearctic and Oriental regions.

#### Biology.

Unknown.

### Key to known species of *Myiocephalus* Marshall

**Table:** 

1	Laterope, on the basal half of the first metasomal tergite, visible in dorsal view (Fig. 10); occipital carina reaching dorsally near upper level of eye and sinuate laterally (Fig. 5)	**2**
–	Laterope, on basal half of first metasomal tergite, not visible in dorsal view (Fig. 14); occipital carina dorsally distinctly below upper level of eye and straight laterally (Fig. 15); West Palaearctic: Austria, Belarus, Bulgaria, Czech Republic, Italy, Netherlands, NW Russia; East Palaearctic: China (Shaanxi), Far East Russia	***M. niger* Fischer, 1957**
2	Area near occipital carina and occiput dorsally pale yellow (Fig. 16)	**3**
–	Area near occipital carina and occiput dorsally reddish brown (Fig. 3) or dark brown (Fig. 19)	**4**
3	The scapus of ♂ 1.0 × as long as wide (Fig. 17); minimum width of face 2.0 × as long as height (Fig. 17); length of the malar space of ♂ 1.1 × basal width of the mandible (Fig. 17); Worldwide (China (Heilongjiang, ^1^Jilin (Mt. Changbai), Taiwan)	***M. boops* (Wesmael, 1835)**
–	The scapus of ♂ 1.3 × as long as wide (Fig. 18); minimum width of face 1.6 × as long as height (Fig. 18); length of the malar space of ♂ 1.2 × basal width of the mandible (Fig. 18); Nearctic: Canada, USA	***M. laticeps* (Provancher, 1886)**
4	Area near occipital carina dark brown and occiput dorsally brown (Fig. 19); vein cu-a of fore wing of ♀ as long as 1-CU1 and oblique (Fig. 20); prepectal carina absent medio-ventrally (Fig. 21); antenna of ♀ with 29 segments; West Palaearctic: Austria	***M. zwakhalsi* van Achterberg, 2019**
–	Area near occipital carina and occiput dorsally reddish brown (Fig. 3); vein cu-a of fore wing of ♀ distinctly longer than 1-CU1 and vertical (Fig. 7); prepectal carina present medio-ventrally (Fig. 8); antenna of ♀ with 32 segments (Fig. 6); East Palaearctic: China (Ningxia, Hubei)	***M. cracentis* Li, sp. nov**.

### Myiocephalus cracentis

Taxon classificationAnimaliaHymenopteraBraconidae

Li sp. nov.

F4C11AD1-992A-59E6-8E58-4F408D71C8D0

http://zoobank.org/A97765F9-4219-40D9-B5EF-1D08394FCA68

Figures 1–2 , 3–11 , 12–13

#### Type material.

***Holotype*,** ♀, NW China, Ningxia Province, Liupanshui, Liangdianxia, 21.viii.2001, Guang-hong Liang.

***Paratypes*:** 1♀, same label data as holotype; 1 ♂, C China, Hubei Province, Shennongjia, Tianmenya, 17. viii. 1988, Juchang Huang.

#### Description.

Holotype, ♀, length of fore wing 3.4 mm, and of body 3.7 mm.

***Head*.** Antenna with 32 segments and 1.2 × as long as fore wing, third segment 1.1 × as long as fourth segment, third, fourth and penultimate segments 4.6, 3.9 and 2.8 × as long as wide, respectively (Fig. 6); eye 3.4 × as long as temple in dorsal view; temples directly and linearly narrowed behind eyes (Fig. 3); OOL:OD:POL = 8:4:13; vertex and frons largely superficially coriaceous and shiny; in front of anterior ocellus with small convexity (Fig. 3); occipital carina complete and dorsally remaining shortly below upper level of eyes (Fig. 5); minimum width of face 1.9 × as long as height; face mainly very finely densely punctulate, but latero-ventrally largely smooth, with whitish setae and satin sheen (Fig. 4); clypeus convex medially and with slightly concave and thin ventral lamella (Fig. 4), medially finely rugulose; anterior tentorial pits large (Fig. 4); malar suture deep, narrow and straight; length of malar space equal to basal width of mandible and malar space in anterior view straight (Fig. 4); mandible slender, strongly twisted, outer side convex and with deep basal depression (Fig. 4), its second tooth similar to first tooth and acute.

**Figures 1, 2.**
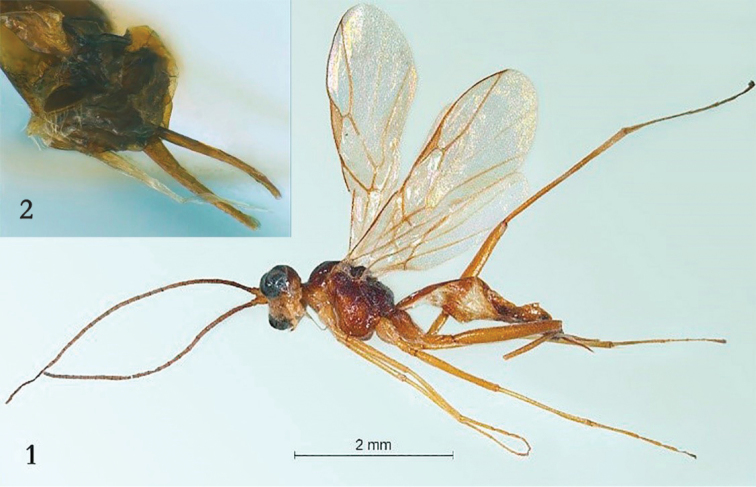
*Myiocephalus cracentis* sp. nov., ♀, holotype. **1** Habitus, lateral aspect **2** ovipositor and its sheath, lateral aspect.

***Mesosoma*.** Length of mesosoma 1.3 × its height; side of pronotum mainly rugulose, dorsally largely punctulate (Fig. 8); mesopleuron dorsally densely and finely rugulose (Fig. 8), ventrally largely (including precoxal sulcus) rugose; prepectal carina completely present; episternal scrobe linear, long and posteriorly deep (Fig. 8); mesosternum sparsely setose, convex and shiny; mesosternal suture shallow, narrow and smooth; notauli absent, mesoscutum sparsely setose, flattened, moderately shiny, and its posterior half with posteriorly converging aciculation (Fig. 9); scutellar sulcus smooth and deep (Fig. 9); scutellum anteriorly convex, rugulose (except some rugae antero-laterally) and shiny, medial part coriaceous, medio-posteriorly convex, smooth and with no depression (Fig. 9); metapleuron coriaceous-rugulose (Fig. 8); propodeum rectangularly depressed medially (Fig. 10).

**Figures 3–11.**
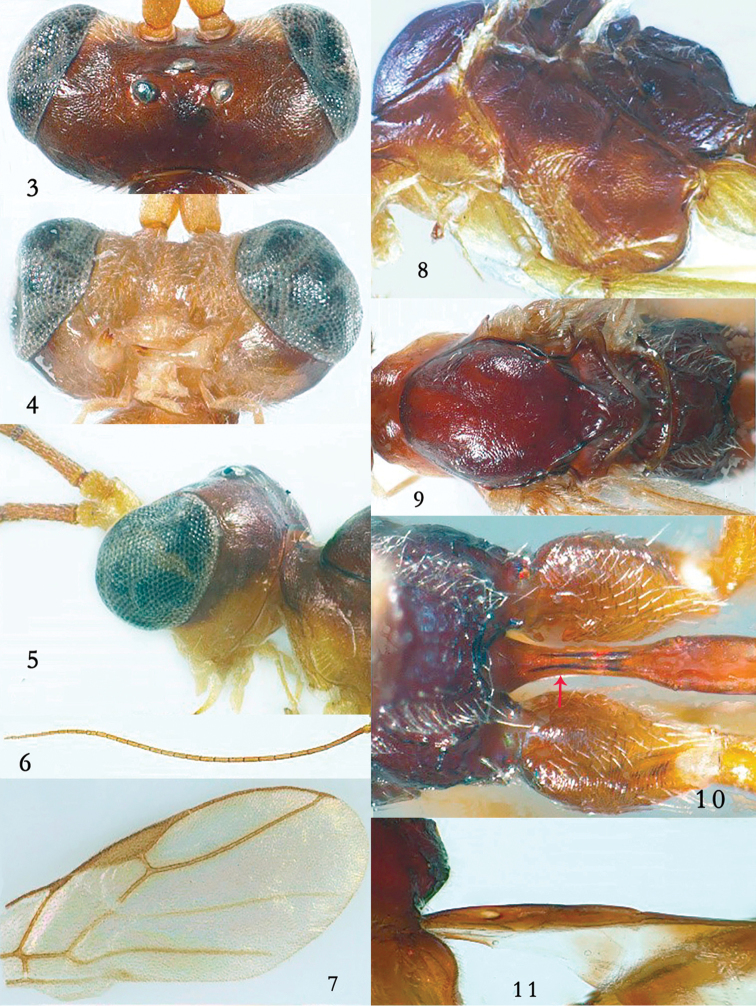
*Myiocephalus cracentis* sp. nov., ♀, holotype. **3** Head, dorsal aspect **4** head, anterior aspect **5** head, lateral aspect **6** antenna **7** fore wing **8** mesosoma, lateral aspect **9** mesosoma, dorsal aspect **10** propodeum and first metasomal tergites, dorsal aspect **11** first metasomal tergite, lateral aspect.

***Wings*.** Fore wing: 2-M unsclerotized; 1-R1 1.1× longer than pterostigma; marginal cell slender; r:3-SR+SR1:2-SR = 1:11:3; vein r issued from middle of pterostigma; vein SR1 very slightly curved basally (Fig. 7); 1-CU1 slender and vertical; cu-a vertical and distinctly longer than 1-CU1; 1-CU1:2-CU1 = 3:13; basal and subbasal cells of fore wing similarly setose as other cells. Hind wing: M+CU:1-M:1r-m = 27:5:7; basal 0.7 of M+CU unsclerotized.

***Legs*.** Middle and hind legs very slender tibia and tarsus together ca. 2.4 × longer than femur, tibia ca. 3.7 × longer than coxa; fore leg normal, tibia nearly 3 × as long as coxa; length of femur, tibia and basitarsus of hind leg 7.6, 22.7 and 6.0 × as long as their maximum width; hind tibial spurs 0.2 × as long as basitarsus.

***Metasoma*.** First tergite 5.1 × longer than its maximum width, basal half with distinctly concave sides and laterope partly visible, distinctly widened basally, flat (except minute depression near adductor) and smooth; in lateral view slender, posterior half convex, subparallel-sided (Fig. 10); first tergite open ventrally and laterope very deep and large (Fig. 11); following segments smooth, compressed and shiny, tergite three to eight concave medio-apically; second metasomal suture distinct; sternites folded medially; hypopygium folded and sclerotized medially, protruding medio-posteriorly and with apical fringe of bristly setae (Fig. 2); ovipositor sheath robust, parallel-sided, widened basally and slight widened apically, its setose part 0.2 × as long as fore wing (but dorso-basally glabrous) and ca. 5.8 × longer than its basal width; lower valve of ovipositor compressed, widened in lateral view and apex of upper valve narrow and remainder cylindrical (Fig. 2).

***Color*.** Reddish brown, dorsally dark than ventrally; palpi, mandible, scapus, pedicellus ventrally, face, tegula, fore leg (but tarsus largely, femur and trochantellus partly infuscate), pronotal side ventrally, propleuron, mesosternum anteriorly, middle leg (except dark brown trochantellus and base of femur), hind tibia and tarsus pale yellowish; pterostigma and most veins of fore wing brown; wing membrane slightly infuscate.

**Figures 12, 13.**
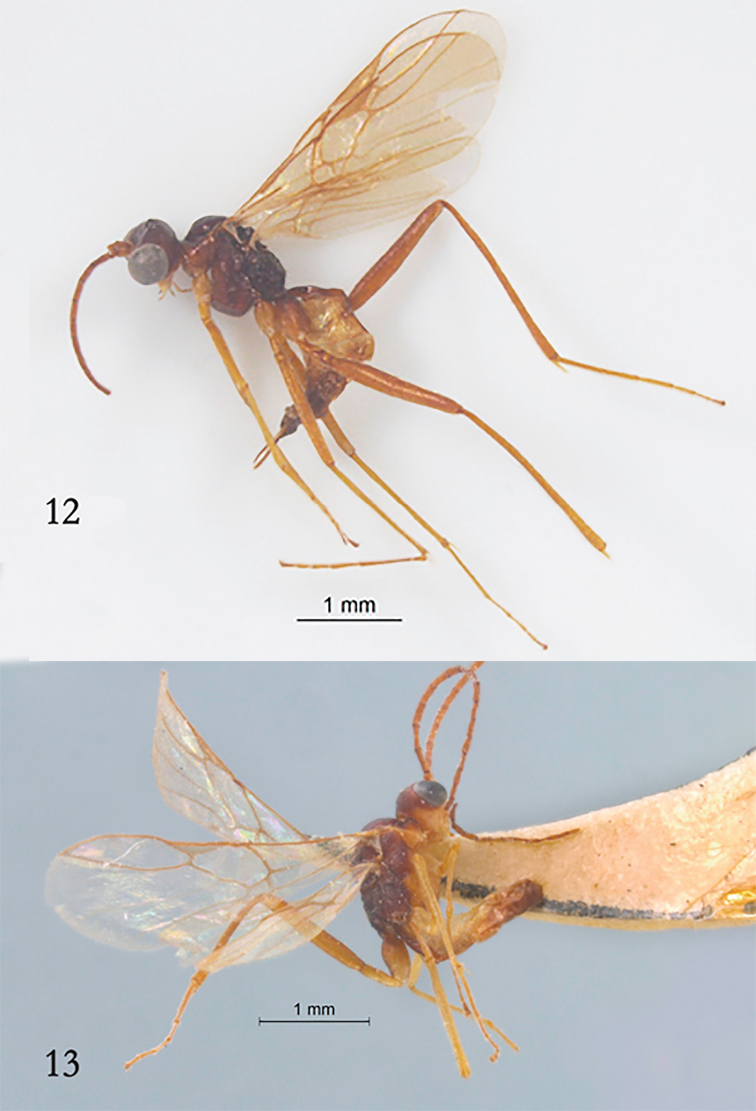
*Myiocephalus cracentis* sp. nov., habitus, lateral aspect. **12** ♀, paratype **13** ♂, paratype.

***Variation*.** Length of fore wing 3.2 mm, and of body 3.5 mm (Fig. 12).

**Male.** Length of fore wing 3.0 mm, and of body 2.9 mm; antenna with 30 segments; length of malar space 1.8 × basal width of mandible; first tergite smooth and shiny; only sternites of basal half of metasoma folded medially and tergite three to eight weakly concave posteriorly (Fig. 13).

#### Remarks.

The new species runs in the key by Tan et al. (2019) to *M. zwakhalsi*, but differs from it as follows: 1) vein cu-a of fore wing distinctly longer than 1-CU1 and vertical (ca. as long as 1-CU1 and oblique in *M. zwakhalsi*); 2) prepectal carina present medio-ventrally (absent medio-ventrally); 3) scapus yellow dorsally (dark brown); 4) first metasomal tergite of ♀ slender in lateral view (robust); 5) hind coxa rugulose-striate (finely striate); 6) setose part of ovipositor sheath ca. 5.8 × longer than its basal width (ca. 4.2 × longer than its basal width).

#### Biology.

Unknown.

#### Distribution.

China (East Palaearctic).

#### Etymology.

Named after the slender pterostigma and marginal cell of the fore wing, long narrow legs, and antennae: “cracentis” is Latin for “slender, graceful”.

**Figures 14–19.**
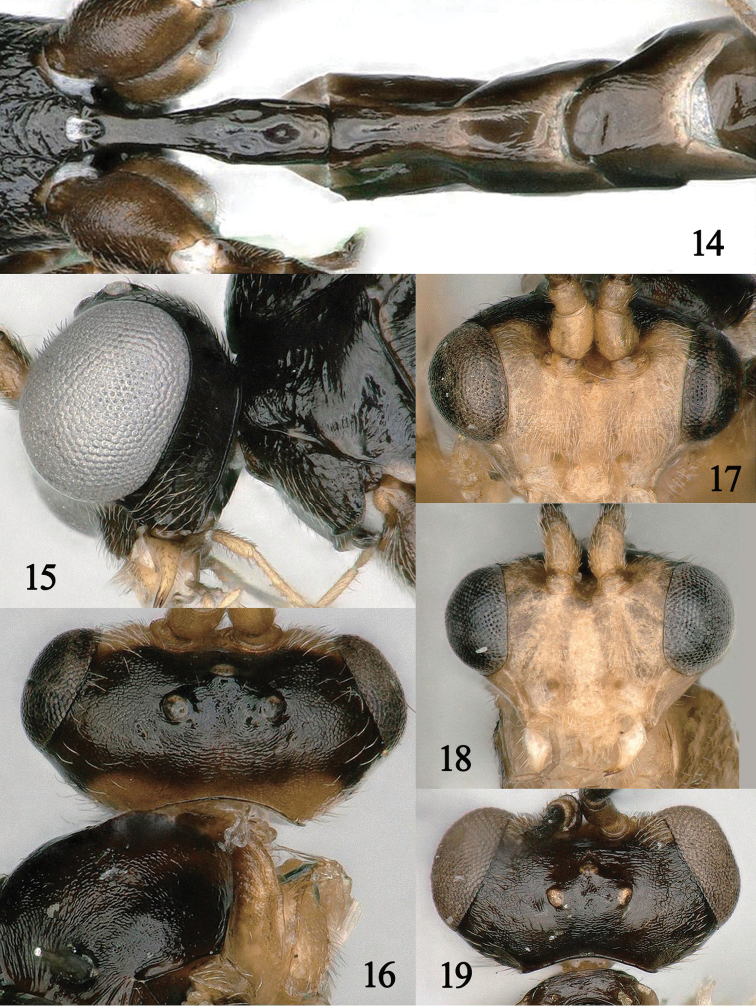
**14, 15***Myiocephalus niger* Fischer, ♀ **14** first metasomal tergites, dorsal aspect **15** head, lateral aspect **16, 17***Myiocephalus boops* (Wesmael), ♂ **16** head, dorsal aspect **17** head, anterior aspect **18***Myiocephalus laticeps* (Provancher), ♂, head, anterior aspect **19***Myiocephalus zwakhalsi* van Achterberg, ♀, head, dorsal aspect (figures 14–19 from Tan et al. 2019).

**Figures 20, 21.**
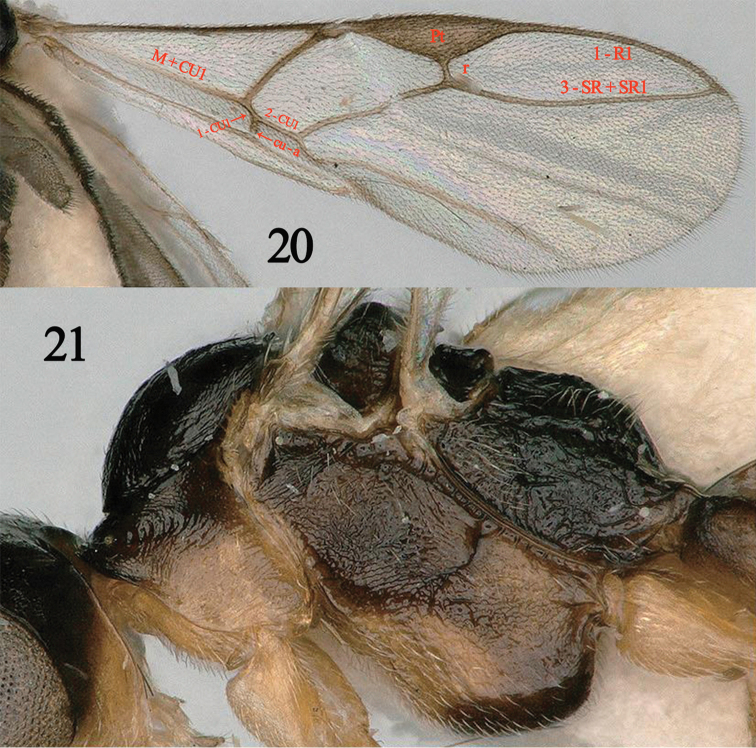
*Myiocephalus zwakhalsi* van Achterberg, ♀. **20** Fore wing **21** mesosoma, lateral aspect (from Tan et al. 2019).

## Supplementary Material

XML Treatment for Myiocephalus

XML Treatment for Myiocephalus cracentis

## Footnotes

1 Represents a new record
